# Residency training in the time of COVID-19: A framework for academic medical centers dealing with the pandemic

**DOI:** 10.1007/s40037-020-00622-z

**Published:** 2020-10-09

**Authors:** Sawsan Abdel-Razig, Waqaas Ahmad, Mahdi A. Shkoukani, Ahmad Nusair, Antonio Ramirez, Kashif Siddiqi, Yasir Akmal, Ziyana Al Khusaibi, E. Murat Tuzcu

**Affiliations:** 1 Cleveland Clinic Abu Dhabi, Abu Dhabi, United Arab Emirates; 2 Cleveland Clinic Lerner College of Medicine, Case Western Reserve University, Cleveland, OH USA

**Keywords:** COVID-19, Coronavirus, Pandemic, Graduate medical education, International medical education, Academic medical center, Academic health center

## Abstract

**Background:**

As cases of COVID-19 climb worldwide, academic medical centers (AMCs) are scrambling to balance the increasing demand for medical services while maintaining safe learning environments. The scale and nature of the current pandemic, limitations on key resources, risks of transmission, and the impact on trainee wellbeing pose additional challenges to AMCs. We propose a framework for AMCs to utilize in facilitating health system, organization and program-level adjustments to meet the needs of medical trainees during the pandemic.

**Approach:**

In February 2020, we developed a three-level approach to the pandemic response of training programs at our AMC. The first level involved AMC alignment and engagement with regulatory stakeholders. The second level utilized the graduate medical education committee and leveraged organizational functions such as human resources, finance, and clinical departments. The third level of intervention focused on common approaches used by programs to ensure continuity of learning in the context of dynamic changes in workflows and service operations.

**Evaluation:**

Outcomes at each level are reported. These include the co-development of a national framework on medical trainee responses to COVID-19, the composition of an operational guidance document, organizational protocols to accommodate novel challenges posed by the pandemic, and multiple program-level interventions.

**Reflection:**

This methodical approach, employed during a global crisis, was critical in facilitating interventions required to fulfill the mission of AMCs. Future steps include assessing the impact of these changes on trainee performance and the applicability of the approach in diverse settings.

**Electronic supplementary material:**

The online version of this article (10.1007/s40037-020-00622-z) contains supplementary material, which is available to authorized users.

## Background and need for innovation

There are over 30 million cases of COVID-19 worldwide [1]. Academic medical centers (AMCs) serve as significant players in the global response to the evolving pandemic. Accordingly, medical trainees have been increasingly recognized as a critical contingency of frontline healthcare professionals. Multiple educational stakeholders have called for extraordinary provisions to facilitate their deployment into the workforce [2, 3] while heeding the need to safeguard their wellbeing and assuring the continuity of their educational programs [4, 5]. This has left AMCs to determine how to balance increasing demands on medical services while maintaining safe learning environments that provide meaningful educational experiences. Put to task, medical educators worldwide have shared their experiences on the pandemic responses of their respective AMCs with insights primarily falling along three main axes: clinical service provision, preserving resident health, and educational continuity [6–10]. Moreover, an expanding body of literature on innovative educational strategies, trainee perspectives, and personal reflections of educational leaders has further enriched our understanding of the scale and nature of challenges faced by AMC’s [11–17]. Nonetheless, most studies have focused on single program or institutional interventions, with a relative scarcity of information on whole system approaches that encompass regulatory, operational, and academic stakeholders.

## Goal of innovation

We set out to develop a multi-level, systematic framework towards the development and application of graduate medical education (GME) interventions in the context of the pandemic. More specifically, we describe an approach utilized by our AMC (the Cleveland Clinic Abu Dhabi) in facilitating health system, organization and program-level adjustments to meet the needs of graduate medical trainees during pandemic situations.

## Steps taken for development and implementation of innovation

Cleveland Clinic Abu Dhabi operates eight GME programs spanning surgical and medical specialties and serves as a quaternary referral center for the country and the region. During the pandemic, our institution was one of several COVID-19 designated facilities (as determined by the national authorities for emergency response and crisis management). As the pandemic progressed, increasing demand for these services led to significant shifts in clinical workflows, redeployment of non-trainee faculty/staff, the interruption of clinical services such as elective surgeries, and a surge in inpatient, critical care, and employee-related health services. Timely, effective, and coordinated, national pandemic responses including the expansion of non-hospital-based COVID-19 care and isolation settings, facilitation of widespread testing and contact tracing, and adequate procurement and stockpiling of personal protective equipment significantly facilitated our AMC’s ability to continue providing care without exceeding surge capacity or increasing reliance on residents. Nonetheless residents were significantly impacted by changes to faculty clinical responsibilities, operational restructuring, and experience with potential or confirmed COVID-19 patients.

In February 2020, we initiated a three-level approach to the pandemic response of training programs at our AMC. Recognizing the importance of having a cohesive national response, it was evident that the usual institution-centric approach would not address the novel yet universal challenges presented by the pandemic. The first level required system interventions including early engagement and alignment with local and international regulators of GME including all national health departments/authorities, licensing bodies, accreditors, board certifying organizations, and other GME institutions. The Designated Institutional Official at Cleveland Clinic Abu Dhabi called for meetings to understand the operational challenges faced by each entity, contingencies being planned, positions taken and sought overall guidance. These meetings consisted of brief presentations from each designated institutional official on institutional interventions, areas of innovation and challenges, with specific focus on opportunities for resource pooling. Meetings focused on the formulation of consensus guidelines and protocols based on proceedings and discussions.

The second level involved a departure from the usual academic focus of institutional residency governance committees to include complementary organizational functions. The GME committee convened in a series of emergency meetings with organizational departments, such as finance, human resources and legal, to collectively review organization’s responses to the pandemic and their impact on GME programs. At each meeting, program directors presented their insights on areas impacting their programs including national and organizational mandates related to the pandemic, changes to clinical workflow in their department and administrative activities, and personal/social issues affecting their residents. The focus of discussions included identification of organizational resources that may be available to programs and the consideration of exceptional organizational provisions to cater to the unprecedented needs of programs and residents.

The third level of intervention consisted of amendments to clinical training programs specifically focused on identifying common approaches to pandemic-induced changes in workflows, service operations, and mental and physical health needs of medical residents and fellows. Far from the usual specialty centric approach to teaching and learning, these processes facilitated the identification of common projects that could be implemented across the differing specialties, and the recognition of specialized skill sets or resources that may be shared amongst the programs. Additionally, there was a need to fill sudden gaps in educational experiences with substantive teaching due to a significant pandemic-driven reduction in service lines. Even in areas that maintained high patient volumes, such as the emergency department, inpatient medicine, and intensive care units, a scarcity of available safe clinical encounters for trainees and the need to preserve personal protective equipment and reduce contact exposure in high risk COVID-19 areas led to a significant reduction in clinical bedside teaching opportunities.

**Fig. 1:**
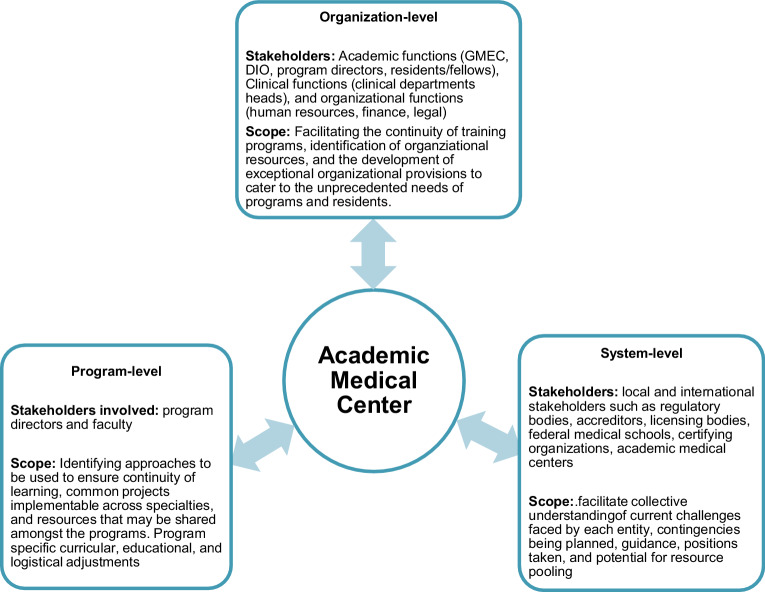
Systematic approach to GME responses to the pandemic: stakeholders, scope, and the role of the AMC

Fig. 1 summarizes the processes utilized at each level and the central role of the AMC in facilitating them. It is essential that central and bidirectional channels of communication are established between levels, representing a critical role of AMCs.

## Outcomes of innovation

Objectives and outcomes at each level are outlined in Tab. 1. On the system level, the primary outcomes from the engagement of educational stakeholders at regulatory and operational levels across the nation was the development of the first national GME standards in response to the pandemic (Supplement 1 of the online Supplementary Material). This document provided foundational recommendations and exceptional regulatory provisions for medical trainees and training centers dealing with the pandemic. The contents of this consensus guidance document included pivotal direction to AMCs dealing with the pandemic and served to facilitate the development of accompanying operational processes, protocols and strategies that align with the regulatory standards.

**Table 1:** Objectives and outcomes achieved at each level of GME intervention

	Objective	Example outcome
*System*	– Development of a national, unified, regulatory consensus framework on graduate medical education pandemic response including input from all relevant stakeholders (licensing bodies, accreditors, certifying agencies, AMCs)	– Publication and/or dissemination of national/local pandemic response standards
	– Operational guidance for AMCs to facilitate alignment with regulatory framework (DIO expert consensus)	– Development and dissemination of institutional protocols that align with regulatory framework
*Organization*	– Mobilization of cross-specialty efforts including joint teaching opportunities, consensus on curricular modification strategies, and identification of appropriate opportunities for trainee placement in organizational pandemic related responses	– Launch of online cross-specialty curriculum to provide additional learning experiences for programs with reductions in safe clinical learning opportunities due to the pandemic
		– Identification of organization-wide, non-patient facing, pandemic response functions that residents where residents may be allocated to count towards curricular elective or core experiences
		– Organization wide cessation of clinical elective experiences to mitigate exposure risk in non-essential rotations
		– Deployment of curricular modifications such as changes to minimal on-call schedule requirements to enable decreased resident deployment in high-transmission risk learning environments (e.g. ICUs)
	– Engagement of non-academic functions (e.g. HR, finance, legal) to enhance logistical support and identification of pandemic-related organizational protocols/policies/needs that affect medical trainees	– Identification of impact of social distancing measures on resident facility space needs (call rooms, lounges, work spaces) with compensatory space provided
		– Roll out of additional housing provisions to cater to residents living at a distance from the facility due to pandemic imposed travel restrictions
		– Execution of emergency evacuation/repatriation processes trainees abroad at the time of the pandemic
		– Review and assurance of health and mental health coverage for COVID-19 related resident needs
		– Review and execution of screening and quarantine protocols for COVID-19 afflicted residents
*Program*	– Utilization of online platforms for the delivery of educational content	– Morning reports, educational conferences, morbidity and mortality rounds, journal clubs, etc. all held online
	– Development and delivery of educational content on pandemic related information	– Teaching sessions on donning and doffing PPE, medical/surgical management of COVID-19 patients using online learning management systems
	– Addressing resident wellbeing	– Regular small group discussions and lectures held online between program faculty and residents with topics including stress management, mental health, resident led wellbeing topics, etc
		– Dissemination of information to residents on institutional resources for well being
		– Augmenting access and availability of occupational/mental health services for residents
	– Altering resident scheduling to adjust for shifting service line volumes, workflows, availability of resources and the learning environment	– Reallocation of residents on elective, off-site, and ambulatory experiences to inpatient rotations, COVID-19 related administrative/non-patient facing tasks such as calling families, contract tracing, etc
	– Flexibility in curricular time to accommodate significant clinical service interruption	– Shifting of curriculum to advance quality improvement, patient safety, clinical research, volunteer work, and other forms of scholarly activity/experiences while delaying clinical rotations towards later in the academic year
	– Continuous considerations and adjustments of day-to-day clinical tasks assigned to medical trainees with specific consideration of PPE stewardship, availability of appropriate supervision, and task relevance to educational objectives	– Modifying clinical rounding practices to mitigate exposure risk to residents
		– Shifting personal encounters with patients to telehealth where appropriate and available
		– Minimizing resident involvement in emergency surgery/procedures

*AMC* academic medical center*, GME* graduate medical education,* DIO* designated institutional official, *HR* human resources *ICUs* intensive care units, *PPE* personal protective equipment

On the organization level, the approach mentioned above served to galvanize all AMC functions towards the unified goal of upholding our academic mission despite extreme circumstances, identify consensus strategies to be used, and recognize inter-departmental resources. Resultant outcomes focused on two general areas, joint curricular efforts and additional organizational logistical support to trainees to meet their needs during the pandemic. An example of curricular efforts included the immediate mobilization of faculty to develop a cross-specialty, on-line, didactic course to address the lost curricular time due to the disruption of safe clinical learning experiences. This core series was made available to all program directors and faculty to incorporate within their curricula as they saw fit. In terms of logistical support, these outcomes included issues of physical space at work, health and mental health coverage, as well as travel, housing, and other areas impacted by the pandemic. For example, a critical component included the identification of immediate modifications to resident work and communal spaces to accommodate for social distancing requirements. These were promptly flagged by facility operations teams, converted to the appropriate maximal occupancy, and most importantly, compensatory resident space provisions were incorporated into emergent pandemic-related facility outfits.

At the program level, initiatives differed due to the variable impact of the pandemic on each program, but fell along six broad categories of objectives and outcomes as described in Tab. 1.

## Critical reflection

We describe a systematic approach used by our AMC to address pandemic-related changes to GME. We believe the methodology behind this tiered approach is applicable to differing contexts. Though setting-specific stakeholders, strategies, and operations may exist, the general objectives and methodical approach described are likely useful to institutional and educational leaders globally. This is supported by recent studies where similar approaches have been instituted [10, 18].

As described earlier, centralized, top heavy, streamlined approaches to pandemic response management have been effective in the UAE, which has one of the lowest COVID-19 case fatality rates in the world [19]. When reflecting on our experience in implementing a similarly centralized approach to the GME response to the pandemic, unification of system-level objectives, served to align all relevant entities, facilitate the use of resources between entities, and anticipate downstream effects of pandemic-related interventions on the educational system as a whole. Activities on this level were facilitated with stakeholders quickly coming to consensus on priorities, willfully sharing areas of challenge, and identifying resources to pool when applicable. This is likely due to the relatively small numbers of educational stakeholders (allowing for personal familiarity and interactions) as well as the traditionally centralized governing structures of the health systems. Hence our methodology may be particularly useful to countries with similar regulatory and demographic characteristics.

On the organizational level, initial challenges included familiarizing business functions that do not usually have a significant role in GME with the needs of trainees in the pandemic and their respective roles in addressing them. Moreover, ensuring consideration of GME needs within rapidly evolving, pandemic-related, organizational decision-making was critical and was sometimes met with pushback due to competing organizational priorities (for example deciding on space allocations for trainees versus patient care). Having GME leaders on hospital-related COVID-19 committees/taskforces and providing anticipatory guidance to faculty and program directors in rapidly identifying potential impact of organizational decisions proved useful.

On the program level, there were several central issues that most programs faced: 1) balancing service and learning needs, 2) educational continuity for trainees while mitigating risk exposure, 3) addressing unique physical and mental health needs spurred by the pandemic, as described by colleagues worldwide [9, 10]. Universal efforts such as joint curricular initiatives, leveraging virtual/online learning opportunities, and nimble rotation adjustments were key in maintaining educational continuity. However, when considering exposure mitigation and service vs. teaching balance, program/rotation specific changes were more effective. For example, in some wards, rounds were converted to virtual platforms where residents continued to lead them and follow up on non-patient facing clinical tasks while in-person rounds were limited to attendings.

Though certainly there is no one-size-fits-all solution, there does seem to be a universal observation from our experience: the importance of consistent and frequent forums for convening. This is supported by recent literature where managing communication and maintaining a sense of community were identified as key components to the successful pandemic response of AMCs [14, 18, 20]. Connecting in person, even if virtually, was critical for both faculty and residents. With faculty, we used the GME committee meetings as the centralized venue for the exchange of ideas across programs and sharing of individual challenges. This served to foster a sense of shared purpose, identify areas for joint efforts, and provided an opportunity to learn from each other. For residents, program directors and the designated institutional official initiated regular group meetings with all residents. These meetings addressed concerns while providing important venues for residents to learn about organization and system level changes (for example, changes in national travel advisories). Anecdotally, residents reported that access to this information via in-person meetings with faculty and peers, even if not relevant to their day-to-day clinical duties, helped ease stress, improve connectedness, and provided a sense of shared experience.

The primary limitation of our framework is that it does not address issues raised by health systems faced with ethical concerns of trainee deployment in the pandemic workforce, [2–5, 15] as this was not relevant in our context. However, other common ethical considerations such as the significant challenges around balancing service and education demands and issues of learning continuity in pandemic conditions are the focal points of the framework presented [9, 10, 13, 14]. The inability to assess the impact of these interventions on resident performance also presents a challenge. Such information may be best analyzed using modified formative and summative assessment frameworks and high stakes evaluation strategies such as in-training exam performance and board pass rates for cohorts affected by this pandemic. Additionally, we have not yet formally assessed resident satisfaction and perceptions during this time period; however, recent literature from medical trainees aligns with changes suggested by our model [14, 16].

## Caption Electronic Supplementary Material

Medical Education Standards in response to COVID-19 (UAE National Institute for Health Specialities)
