# Supplementary material for: Residency training in the time of COVID-19: A framework for academic medical centers dealing with the pandemic
Source: Perspect Med Educ. 2020 Oct 9;10(2):135–40. doi: 10.1007/s40037-020-00622-z (PMC7545371; doi:10.1007/s40037-020-00622-z)
Supplement: Supplementary file 1 — Medical Education Standards in response to COVID-19 (UAE National Institute for Health Specialities) [file 40037_2020_622_MOESM1_ESM.pdf]

United Arab Emirates

المعهد الوطني للتخصصات الصحية

Medical Education Standards in response to COVID-19

1. **Rationale:**

   As the COVID-19 pandemic continues to evolve rapidly and the need for physicians and other healthcare professionals to care for patients during this pandemic increases, the National Institute for Health Specialties in collaboration with health regulators in UAE will continue to communicate with training institutions, educators and trainees regularly to inform them of regulatory amendments with respect to medical training programs and trainee requirements to accommodate these unprecedented times.

   Our commitment is facilitate the continued pursuit of medical training and to mitigate interruption of training due to service pressures that may be associated with this global pandemic. We will continuously work with all educational stakeholders including (but not limited to) certifying bodies and accrediting organizations to reach the best outcome possible during this exceptional time.

2. **The Scope of Guideline**

   This guideline is specific to the training period during the COVID-19 pandemic and extends up to 31 Dec 2020 (extendable upon further review) and covers all clinical training programs including internships, residencies and fellowships as well as short term clinical training programs such as clinical attachments/electives, observerships and return to practice training.

3. **Aim**

   To support clinical training program administration and faculty in their academic mission during the COVID-19 pandemic by providing guidance on the maintenance (to the best of their abilities) of the learning environment necessary to attain the educational competencies set forth for their trainees and in light of the current pressures to provide patient care. Additionally, this guideline aims to serve clinical trainees pursuing their educational programs and professionals duties as they manage the impact of these events on their personal and family lives.

4. **Training Academic cycle**

4.1. Trainees who have applied for internships, residencies, and fellowships in the 2020-2021 academic cycle will be interviewed and enrolled as set forth in the respective application processes (TANSEEQ, DRTP, etc). All institutions should complete their selection and match processes prior to 1st of August 2020. All structured programs are scheduled to start on the first week of September 2020.

4.2. Interruption of training is highly discouraged. Trainees who request an interruption of their studies/educational program may compromise the quality of education and negatively impact the training environment. The trainee must inform the program director if interruption occurs due to unforeseen circumstances. The institution maintains the autonomy to decide on whether the trainee may continue the program (as per the relevant human resources policies) and must provide a comprehensive plan for how the loss of educational time will be addressed

4.3. In the case of program closure or interruption the DIO (Designated Institute Officer) or Chair/Chief of academics should communicate with concerned regulatory authority and subsequently the decision will be made to temporarily suspend the program and/or reallocate its trainees to different institution(s). The program must submit all required information on the trainees and program(s) upon the request of the respective regulatory authority.

4.4. Residency and fellowship programs should maintain rotation durations according to accreditation criteria previously set forth. The program may not reduce program length or modify its educational content without making up for missed curricular content and assessments. Internship programs are allowed up to 4 weeks of reduced program duration (and the respective curricular content) as deemed necessary by the institutional academic committee

5. **Training Sites/Departments:**
   5.1. All training facilities and sites that have been accredited and approved by any health regulatory authority within the UAE may be used to train interns, residents and fellows irrespective of the trainee's designated home institution. Decisions on reallocation of clinical trainees to training sites outside their home institutions will be left to the discretion of the home institution and the training site. .
   5.2. Facilities/sites that provide COVID-19 patients care that are present outside the teaching institution may be approved by the institutional academic committee as a teaching site. This includes emergency, inpatient wards, ICUs, consultation services, call centers, triaging centers, testing clinics, and other services.
6. **Supervision:**
   6.1. All medical education training facilities must ensure adequate supervision appropriate to the clinical care delivered and the educational level of the trainee.
   6.2. Medical education preceptors/supervisors are expected to safeguard, to the best of their ability, the health and personal safety of medical trainees and ensure appropriate personnel/staffing, training, educational, equipment and relevant resources are dedicated to protecting trainees involved in patient care activities.
   6.3. Trainees must not be allowed to conduct any clinical or patient care related activities under insufficient supervision.
7. **Medical Trainee Well Being**
   7.1. All medical education training facilities must ensure that the basic needs of medical trainees are provided for adequately including water, food, personal safety, and sleep.
   7.2. All facilities must aim to maintain the required infrastructure and support services to protect trainees from stress and poor mental health during times of crisis. This must include ensuring access to psychosocial and mental health support services, active and regular monitoring of training wellbeing with identification of risks or emerging issues, and the maintenance of an open and supportive environment that encourages communication and allows trainees to express their concerns.

7.3. The aforementioned support must be provided in a manner that respects the trainee's confidentiality and encourages the use of support services when needed.

8. **Work Hour Requirements:**

8.1. All medical trainees must have adequate rest between their clinical duties and duty hours of trainees should be scheduled and monitored in accordance to accreditation standards.

9. **Learning Context:**

9.1. Training programs must, to the best of their abilities, adhere to facilitating and assessing the learning outcomes to assigned clinical rotations. Rotations that take place in other departments, services, or facilities must be assessed and monitored by the PD to ensure appropriate delivery of intended learning objectives.

9.2. Trainees are entrusted to participate in clinical services as assigned by the program and are expected to demonstrate versatility, resilience and a capacity for collaborative teamwork in these extraordinary circumstances.

9.3. Objectives of clinical rotations will be adapted to the level of trainee competence. Trainees will not be asked to undertake tasks for which they do not have adequate supervision

10. **Trainee activities in response to COVID-19**

10.1. All training programs should modify all relevant standards, policies, and protocols to support the recommendations of physical distancing and travel restrictions.

10.2. Activities previously face to face including teaching sessions, administrative meetings, and interviews should be conducted by means of virtual videoconferencing technologies available and should preserve trainee and patient confidentiality and privacy as per convention

10.3. These activities should be carried out to the best of all parties' abilities with goals of preserving quality of content while avoiding subjectivity and bias.

10.4. All electronic activities should comply with UAE standards on electronic communication

11. **Assessment and Evaluation**

11.1. Evaluating the performance of trainees may be challenging due to learning environments affected by the clinical pressures presented by the current global pandemic. Nevertheless, it is imperative to maintain evaluation and performance standards as per accreditation and certification standards for trainees, particularly for final year residents and fellows.

11.2. When applicable, assessments should be redesigned to reflect changes in the curriculum. All forms of formative and summative evaluations shall be conducted whenever it is permissible, utilizing technology means of videoconferencing and electronic evaluation forms.

11.3. For trainees allocated to external (sites outside their home institutions), program directors shall seek the input of the assigned clinical site supervisors, when determining trainee performance.

11.4. The primary responsibility for the oversight of trainee assessment and evaluation lies with the program directors, institutional academic committees, and DIO at all times

11.5. Summative, end-of- year, promotion, advancement, non-advancement, and graduation decisions are left to the discretion of the program directors, institutional academic committees, and DIO. Though these decisions may be made in the absence of high stakes assessments that may have been cancelled or postponed due to the ongoing global pandemic (eg. Board examination results, in-training exams, etc), they must be based on resident performance and other programmatic evaluations available to the institution.

12. **Medical Education Resources**

12.1. Institutions and programs must provide the learning environment resources, facilities, and curricular content to facilitate trainee attainment of educational competencies and skill sets as set forth by accreditation requirements.

12.2. Internship, residency, and fellowship programs across the UAE are encouraged to share educational resources such as curricular content, faculty, staff and administrative resources. Regulatory authorities will facilitate such endeavors when required.

13. **Training program of Observership, clinical attachment and return to practice**

13.1. All short term clinical training programs (electives, observerships, attachments, etc.) requiring physical presence in training facilities should be suspended until further notice.

13.2. COVID-19 voluntary work that is conducted in an authorized regulatory authority may be credited toward training duration for the above programs and is at the discretion of the institutional academic committee. Trainees must get approval from their respective program directors to participate in voluntary work that is to be conducted during curricular time/professional duties.

13.3. Program directors, institutional academic committees, or DIOs will determine if and/or how COVID19 related voluntary work will be counted towards curricular and learning objectives.

14. **Acknowledgment**

14.1. Dr. Farah Al Zaabi, Director Medical Education, Zayed Military Hospital

14.2. Dr. Hatem Faraj Al Ameri, Manager Medical Education and Examination, Department of Health Abu Dhabi.

14.3. Dr. Khalil Ibrahim Qayed, Consultant Training and Development Center, Ministry of Health and Prevention.

14.4. Dr. Mohammed Al Houqani, Secretary General, National Institute of Health Specialties

14.5. Dr. Sawsan Abdel-Razig, Chair Medical Education, Cleveland Clinic Abu Dhabi

14.6. Dr. Wadeia Mohamed Sharief, Director Medical Education and Research Department, Dubai Health Authority.

15. **References**

- “Circular - 17.” Department of Health Abu Dhabi Media Office, 2 Apr. 2020, www.doh.gov.ae/en/resources/Circulars.

- “COVID-19 UPDATE.” ACGME International, 2020, www.acgme-i.org
- “Corona Virus (COVID 19)” Essential Information, 2020, www.mcgill.ca/coronavirus/
- “Advice for Everyone (COVID-19).” NHS Choices, NHS, 2020, www.nhs.uk/conditions/coronavirus-covid-19/
